# Gait video-based prediction of unified Parkinson’s disease rating scale score: a retrospective study

**DOI:** 10.1186/s12883-023-03385-2

**Published:** 2023-10-05

**Authors:** Katsuki Eguchi, Ichigaku Takigawa, Shinichi Shirai, Ikuko Takahashi-Iwata, Masaaki Matsushima, Takahiro Kano, Hiroaki Yaguchi, Ichiro Yabe

**Affiliations:** 1 Department of Neurology, Faculty of Medicine, Graduate School of Medicine, Hokkaido University, Kita 15, Nishi 7, Kita-ku, Sapporo, 060-8638 Hokkaido Japan; 2 RIKEN Center for Advanced Intelligence Project, 1-4-1 Nihonbashi, Chuo-ku, Tokyo, 103- 0027 Japan; 3 Institute for Chemical Reaction Design and Discovery (WPI-ICReDD), Hokkaido University, Kita 21 Nishi 10, Kita-ku, Sapporo, 001-0021 Hokkaido Japan

**Keywords:** Parkinson’s disease, Deep learning, Computer neural networks, Gait analysis, Bradykinesia

## Abstract

**Background:**

The diagnosis of Parkinson’s disease (PD) and evaluation of its symptoms require in-person clinical examination. Remote evaluation of PD symptoms is desirable, especially during a pandemic such as the coronavirus disease 2019 pandemic. One potential method to remotely evaluate PD motor impairments is video-based analysis. In this study, we aimed to assess the feasibility of predicting the Unified Parkinson’s Disease Rating Scale (UPDRS) score from gait videos using a convolutional neural network (CNN) model.

**Methods:**

We retrospectively obtained 737 consecutive gait videos of 74 patients with PD and their corresponding neurologist-rated UPDRS scores. We utilized a CNN model for predicting the total UPDRS part III score and four subscores of axial symptoms (items 27, 28, 29, and 30), bradykinesia (items 23, 24, 25, 26, and 31), rigidity (item 22) and tremor (items 20 and 21). We trained the model on 80% of the gait videos and used 10% of the videos as a validation dataset. We evaluated the predictive performance of the trained model by comparing the model-predicted score with the neurologist-rated score for the remaining 10% of videos (test dataset). We calculated the coefficient of determination (*R*^2^) between those scores to evaluate the model’s goodness of fit.

**Results:**

In the test dataset, the *R*^2^ values between the model-predicted and neurologist-rated values for the total UPDRS part III score and subscores of axial symptoms, bradykinesia, rigidity, and tremor were 0.59, 0.77, 0.56, 0.46, and 0.0, respectively. The performance was relatively low for videos from patients with severe symptoms.

**Conclusions:**

Despite the low predictive performance of the model for the total UPDRS part III score, it demonstrated relatively high performance in predicting subscores of axial symptoms. The model approximately predicted the total UPDRS part III scores of patients with moderate symptoms, but the performance was low for patients with severe symptoms owing to limited data. A larger dataset is needed to improve the model’s performance in clinical settings.

**Supplementary Information:**

The online version contains supplementary material available at 10.1186/s12883-023-03385-2.

## Background

Parkinson’s disease (PD) is the second most common neurodegenerative disease, characterized by bradykinesia, resting tremor, muscle rigidity, and responsiveness to dopaminergic treatment [1, 2]. PD diagnosis and the evaluation of the effectiveness of its treatment require clinical examination. However, owing to the coronavirus disease 2019　pandemic, telemedicine, especially video consultation, has been promoted to reduce the risk of transmission [3, 4]. To further promote the use of telemedicine for PD, the development of methods that can be used to support the evaluation of PD symptoms in telemedicine is needed.

One potential method to evaluate PD motor impairments remotely is video-based analysis [5]. Recently, convolutional neural networks (CNNs), a type of deep learning algorithm, have been used to analyze human actions from videos [6]. However, to date, these models have not been used to evaluate PD symptoms. If these CNN models can be used to evaluate PD symptoms from videos obtained using a standard video camera, remote evaluation through web cameras or smartphone applications, without in-person assessment, may become feasible.

In this study, we focused on gait videos because the gait of patients with PD includes many features characteristic of PD symptoms, such as bradykinesia, shortness of step length, postural abnormality, decreased arm swinging, and freezing of gait. In addition, as the recording of such gait videos does not require specialized skills and is not time-consuming, evaluation of PD symptoms from gait videos would be cost-effective. In both clinical practice and research, the Unified Parkinson’s Disease Rating Scale [7] (UPDRS) is widely used to evaluate PD motor symptoms. Therefore, the aim of this study was to assess the feasibility of predicting UPDRS scores from gait video data of patients with PD using a CNN model.

## Methods

### Patients and video recording

This study included patients with PD who were video recorded from April 2013 to January 2021 while being rated according to the UPDRS [7] for the diagnosis or evaluation of the efficacy of treatment at Hokkaido University Hospital. The diagnosis of PD was made based on the UK Parkinson’s Disease Society Brain Bank criteria [8]. According to the accepted diagnostic criteria, we excluded patients with the following parkinsonian disorders: drug-induced parkinsonism due to dopamine receptor blocking agents; vascular parkinsonism; and atypical forms of parkinsonism, such as progressive supranuclear palsy, multiple system atrophy, or corticobasal degeneration [9]. Additionally, we excluded patients with a history of stroke, hospitalization for a psychiatric disorder, or other neurological, metabolic, or neoplastic disorders, as well as those with symptomatic musculoskeletal diseases such as acute bone fracture, spinal canal stenosis, and osteoarthritis. The Hoehn and Yahr (HY) stages, Mini-Mental State Examination (MMSE) scores, and levodopa equivalent daily dose (LEDD) scores [10, 11] at the first UPDRS assessment were obtained for each patient using their medical records. We also obtained information on whether each patient received device-aided therapies (deep brain stimulation [DBS] or levodopa-carbidopa intestinal gel [LCIG] treatment) from the medical records.

The study protocol was approved by the institutional review board of the Hokkaido University Hospital (approval number: 020–0446), and the requirement for informed consent was waived owing to the retrospective nature of the study. Procedures involving experiments on human participants were performed in accordance with the ethical standards of the Committee on Human Experimentation of the institution in which the experiments were conducted.

We used video data recorded during gait examination to predict the severity of motor symptoms. Videos were captured using a consumer-grade video camera (HDR-XR500V and HDR-CX470B, Sony Corporation, Tokyo, Japan) at 30 fps with a resolution of 1280 × 720 px in MP4 format. In the video recordings, patients wore either a hospital gown provided by the hospital or simple, comfortable clothes. The recordings were conducted in a flat hallway at Hokkaido University Hospital. The camera was placed on a tripod in a fixed location during the recording. The participants were instructed to walk directly toward the camera, turn around, and walk directly away again. Although the walking distance was not predetermined, the patients were instructed to begin walking away from the camera at a distance of 5 to 7 m and then return to a position in front of the camera. They were permitted to use a cane or handrail or to receive assistance from medical staff during the video recording if needed. Tha patients received walking assistance without considering the laterality of their symptoms, and no specific protocol for providing walking assistance was established beforehand. We excluded the video data of patients who walked for less than 10 s and those who could not walk even with assistance (i.e., the score of item 30 of the UPDRS part III was 4). Videos in which the camera was unstable during the recording were also excluded. Whether the video was taken during the medication-on or medication-off phases varied across patients and was not determined in advance. In some cases, certain patients were recorded in both the medication-on and medication-off phases. Video data recorded for the same patient with and without medication and DBS (medication on/off and DBS on/off, respectively) or recorded on different dates were regarded as different videos. We did not repeatedly record videos of the same participant on the same date and during the same treatment state.

### Assessment of UPDRS score

Patients were evaluated using the UPDRS part III at the time of gait analysis, and this examination was included in the video recording. Two experienced movement disorder specialists (T. Kano from April 2013 to June 2017 and S. Shirai from July 2017 to January 2021), both Japanese Society of Neurology-certified neurologists, rated the UPDRS part III score.

### Preprocessing of video data

Ten-second clips were extracted from the recorded videos, specifically those segments where patients began walking. We converted all the frames in the 10 s clips (300 frames per clip) into static images in JPEG format. The static images were resized to 224 × 398 px, and the resized images were cropped to retain only the center 224 × 224 px.

### CNN architecture

**Fig. 1:**
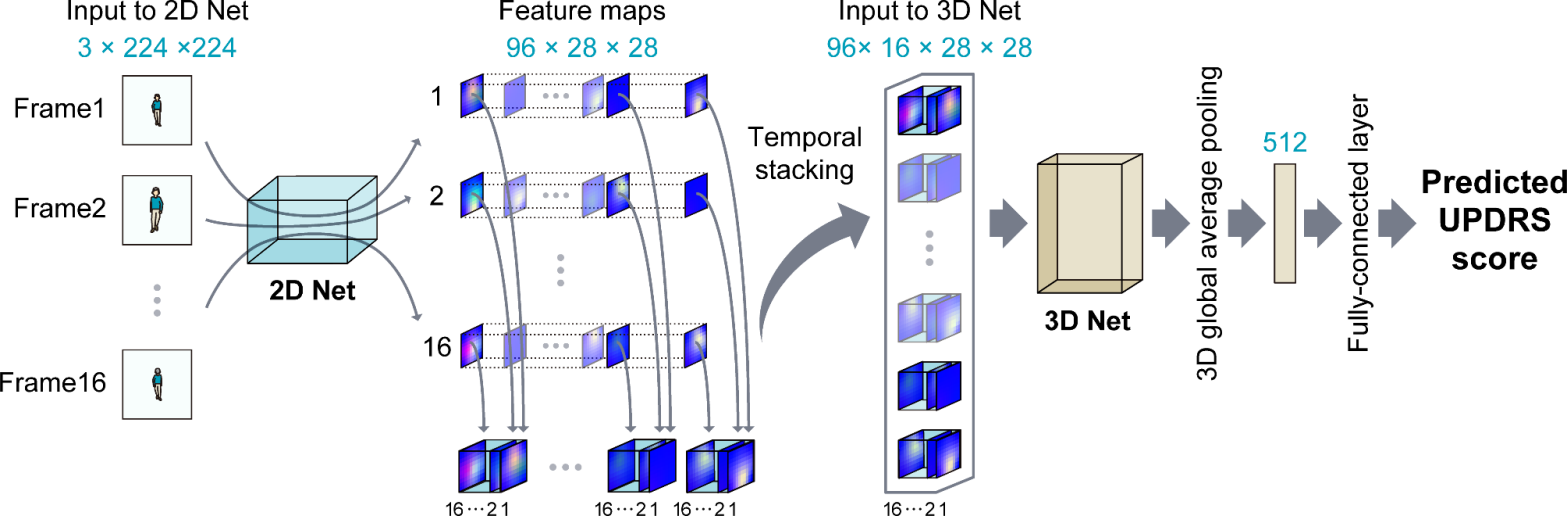
Overview of the ECO-Lite architecture. Input data were RGB color images, 224 × 224 px in size. Sixteen frames were used from each video. The frames were processed using a two-dimensional convolutional network (2D Net) to yield 96 feature maps, 28 × 28 px in size, for each frame. These feature maps were stacked temporally and fed into a three-dimensional convolutional network (3D Net), which was used to analyze the relationships between different frames. Thus, the size of the stacked feature maps used as input was 96 × 16 × 28 × 28. The final output was a predicted score of the Unified Parkinson’s Disease Rating Scale (UPDRS) part III for each video

We used the ECO-Lite CNN architecture [6] in this study, an overview of which is provided in Fig. 1. ECO-Lite is an end-to-end CNN architecture that learns spatiotemporal features from videos. It was originally developed to analyze human action videos and exhibited high performance in classifying 400 human actions in the “Kinetics” video dataset [12]. This CNN model consists of the following two submodules: 2D-Net and 3D-Net. 2D-Net is a neural network with two-dimensional convolutional layers that are used to capture visual features of images from individual frames, whereas 3D-Net has three-dimensional convolutional layers that are used to capture temporal relations between frames.

The input frames were processed with the CNN model, as follows. First, the static frames extracted from videos were provided as input to 2D-Net; second, the output feature maps from 2D-Net were stacked temporally and fed to 3D-Net; and third, the output features from 3D-Net were used for making predictions. For each submodule, we chose the same models as those in the original report [6]: we used a subpart of the “BN-Inception” architecture [13] for 2D-Net and a subpart of the “3D-ResNet18” architecture [14] for 3D-Net, and we attached a fully connected layer for the prediction of the UPDRS score of the input video. We hypothesized that 2D-Net would extract static features of PD, such as postural abnormality, and 3D-Net would extract temporal features, such as walking speed, arm swing, and freezing of gait.

The input data for the model in the original report were 16 frames extracted from each video [6]. Therefore, as input data in this study, we also used 16 color frames, 224 × 224-px in size, extracted at equal intervals from each gait video. The frames were processed using a two-dimensional convolutional network (2D Net) to yield 96 feature maps, 28 × 28 px in size, for each frame. These feature maps were stacked temporally and fed into a three-dimensional convolutional network (3D Net), which was used to analyze the relationships between different frames. Thus, the size of the stacked feature maps used as input was 96 × 16 × 28 × 28. The final output was a predicted score of the UPDRS for each video.

### Model training

At first, we tried to have the model predict the total UPDRS part III score (maximum score: 108) from the gait videos. However, the videos did not contain some aspects of PD symptoms such as voice, tremor, and rigidity, and it would have been difficult for the model to predict the total UPDRS part III score. Therefore, we categorized UPDRS part III into four subscores: axial symptoms, bradykinesia, rigidity, and tremor. The definitions for each subscore were as follows: Axial symptoms included the sum of scores of item 27 (arising from a chair), 28 (posture), 29 (gait), and 30 (postural instability) (with a subscore range of 0–16); bradykinesia consisted of the sum of scores of items 23 (finger taps, total of bilateral hands), 24 (hand movements, total of bilateral hands), 25 (rapid alternating movements of hands, total of bilateral hands), 26 (leg agility, total of bilateral legs), and 31 (body bradykinesia and hypokinesia) (with a score range of 0–36); rigidity was indicated by the score of item 22 (rigidity, total score of head, bilateral hands, and legs) (with a subscore range of 0–20); tremor comprised the sum of scores of items 20 (tremor at rest, total score of head, bilateral hands, and legs) and 21 (action or postural tremor, total of bilateral hands) (with a subscore range of 0–28). We also evaluated the CNN model’s capability to predict these subscores from the gait videos.

In this study, we obtained an average of 10 videos per patient; however, this number greatly varied between patients. Therefore, we divided all video data randomly by stratifying the videos based on the UPDRS score instead of individual patients to create the training, validation, and test datasets (i.e., we permitted videos from the same patient to be in multiple datasets). We stratified the videos into three groups according to the total UPDRS part III score as follows: (1) mild (bottom third), (2) moderate (middle third), and (3) severe (top third). By stratifying the videos according to the UPDRS score, we intended to train the model equally using videos with various severities of PD symptoms. Then, all the video data were randomly divided into 80% (591 videos) for the training dataset, 10% (73 videos) for the validation dataset, and 10% (73 videos) for the test dataset, each with the same proportion of each stratified group. The training dataset was used to train the model, whereas the validation dataset was used to improve hyperparameters, such as the number of training epochs and the learning rate (lr). Finally, we used the test dataset to evaluate the model’s prediction performance using the parameters that showed the best prediction performance in the validation dataset.

The model was trained to predict the total UPDRS part III score and each subscore separately (in this study, we developed five distinct models to predict the total UPDRS part III score and each of the four subscores). The model-predicted scores were compared with neurologists-assigned scores. The prediction errors were calculated as the mean squared error between these two scores. The CNN parameters were updated to reduce the mean squared error in predicting the total UPDRS part III score or the individual four subscores. In the previous report [6], the same model was trained using the “Kinetics” video dataset; therefore, we assumed that the already-trained model could capture basic visual features and their temporal patterns to recognize 400 different human actions. As a result of preliminary evaluations, we implemented a warm-start strategy using the parameter values of this pretrained model as initial values. We fine-tuned those parameters by gradually training submodules with a small lr in the following schedule: 1–20 epochs, only the final fully connected layer was trained with random initialization and lr = 0.001; 21–50 epochs, the fully connected layer and 3D-Net module were trained with lr = 0.0005; 51–70 epochs, all the parameters of the model were trained with lr = 0.0001; and 71–100 epochs, all the parameters of the model were trained with lr = 0.00001. For parameter optimization, we used the Adam optimizer with a weight decay of 0.0005. We set a mini-batch size of 8. We also applied the following data augmentation techniques in every training epoch:

- random horizontal flip (flipping the videos with a 50% probability).
- random rotation (rotating the videos within 5°).
- random color jitter (changing the color values of the videos within 50%).

We used the Python (version 3.8.8) programming language and PyTorch deep learning library (version 1.5.1) [15] to implement, train, and evaluate the CNN models.

### Evaluation of the prediction performance of the model

**Fig. 2:**
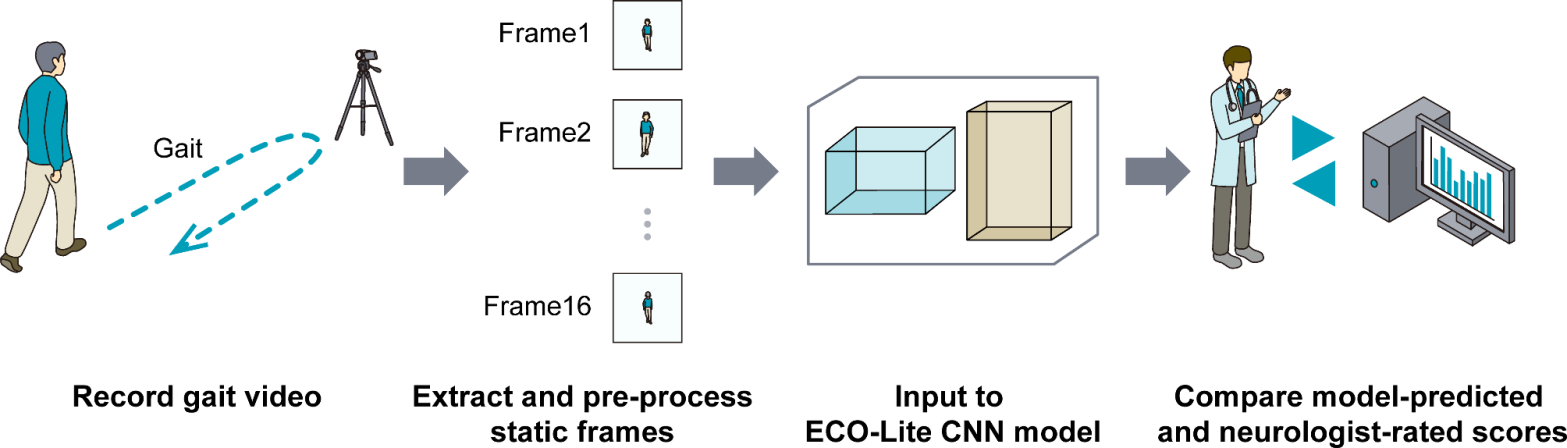
Study workflow. First, we gathered gait videos of patients with Parkinson’s disease, which were recorded using a consumer-grade video camera. Ten-second clips were then cropped from these videos, and 16 static frames were extracted at equal intervals. After preprocessing of the frames, they were inserted into the ECO-Lite convolutional neural network (CNN) model for the prediction of the Unified Parkinson’s Disease Rating Scale (UPDRS) score. The model was trained using training and validation datasets. Finally, we compared the UPDRS scores rated by neurologists and those predicted by the CNN model using the test dataset

The workflow of our prediction evaluation is illustrated in Fig. 2. After the model training described above, we evaluated the prediction performance using the test datasets. For prediction, the output values of the model were rounded to the nearest integers. To assess the disagreement between the model-predicted and neurologist-rated scores, we calculated the mean absolute error (MAE) and the standard deviation (SD). We also calculated the coefficient of determination (*R*^2^) between the model-predicted and neurologist-rated scores to evaluate the goodness of fit of the model for predicting the total UPDRS part III score and each of the four subscores.

## Results

**Fig. 3:**
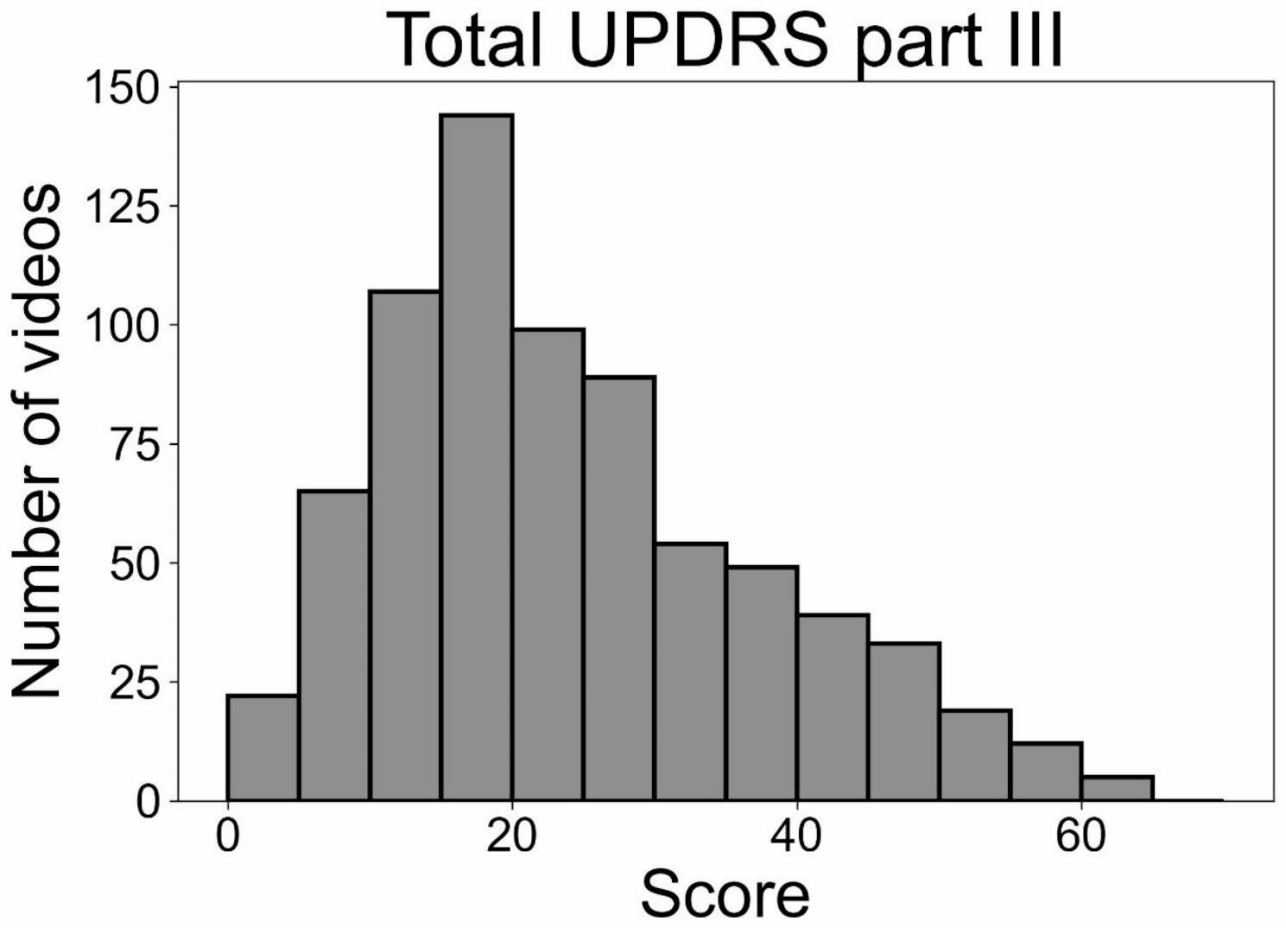
Histogram of the neurologist-rated total UPDRS part III score

**Fig. 4:**
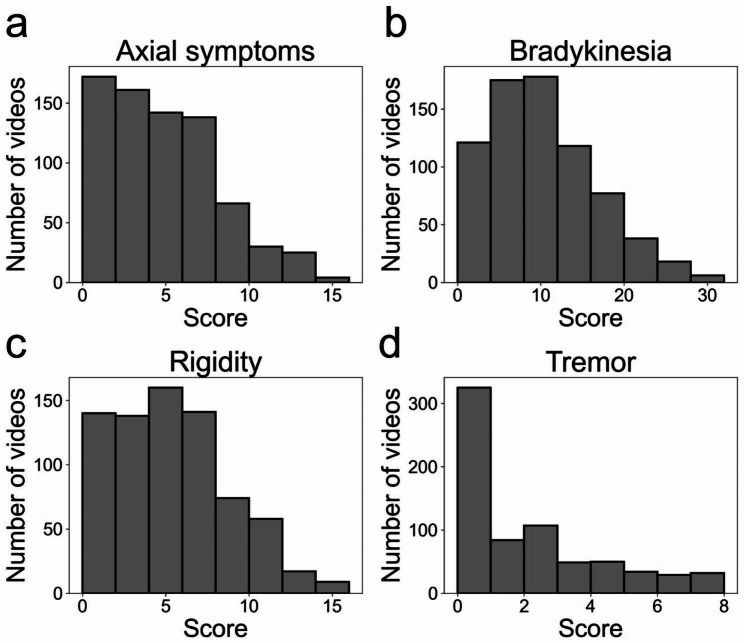
Histogram of the neurologist-rated subscores of the UPDRS part III score. **(a)** Axial symptoms, **(b)** Bradykinesia, **(c)** Rigidity, **(d)** Tremor

During the study period, gait videos of 101 patients were recorded during the UPDRS examination. We excluded 27 patients whose final diagnosis was atypical parkinsonism or vascular parkinsonism. Finally, 737 consecutive gait videos of 74 patients with PD were analyzed in this study (mean, 10.0 ± 10.5 videos per patient). The demographic data of the participants are summarized in Table 1. The mean LEDD ± SD of all participants, except for one patient, at the first UPDRS assessment was 847 ± 444 mg. We excluded one patient from the calculation of mean LEDD owing to the usage of a ropinirole patch, the corresponding LEDD value for which is currently unavailable. Of the 737 analyzed videos, 323 were recorded during the DBS on state, and 2 videos were captured while the patients were undergoing LCIG treatment. There was a total of 72 videos in which the patients used a cane or required assistance during the recording. Additional demographic information for each of the 74 participants is presented in Additional file 1. The mean ± SD neurologist-rated scores for the total UPDRS part III and subscores of axial symptoms, bradykinesia, rigidity, and tremor were 23.7 ± 13.3, 4.2 ± 3.1, 9.8 ± 6.7, 5.3 ± 3.7, and 2.3 ± 2.8, respectively. The distributions of the total UPDRS part III score are illustrated in Fig. 3, while the distributions of the four subscores are depicted in Fig. 4. The total UPDRS score and four subscores of each patient are provided in Additional file 2.

**Table 1:** Demographic data of participants (N = 74)

Characteristics	Data
Women, n (%)	44 (59)
Men, n (%)	30 (41)
Age at first video assessment, years, mean ± SD	63.4 ± 8.2
Disease duration at first video assessment, years, mean ± SD	11.3 ± 5.0
HY stage at the medication-on state at first video assessment, mean ± SD	2.6 ± 0.8
HY stage at the medication-off state at first video assessment, mean ± SD	3.7 ± 1.1
MMSE score at first video assessment^a^, mean ± SD	27.9 ± 2.0
LEDD score at first video assessment^b^, mg, mean ± SD	847 ± 444
Patients who underwent DBS, n (%), mg, mean ± SD	42 (57%)
Patients who received LCIG, n (%)	2 (2.7%)
Patients who required assistance or used a cane at least once during the video recording, n (%)	31 (41.9%)

SD, standard deviation; HY stage, Hoehn and Yahr stage; MMSE, Mini-Mental State Examination; LEDD, levodopa equivalent daily dose; DBS, deep brain stimulation; LCIG, levodopa-carbidopa intestinal gel.

^a^Two patients were not assessed via the MMSE.

^b^One patient was excluded from the calculation of LEDD because of the usage of a ropinirole patch.

**Fig. 5:**
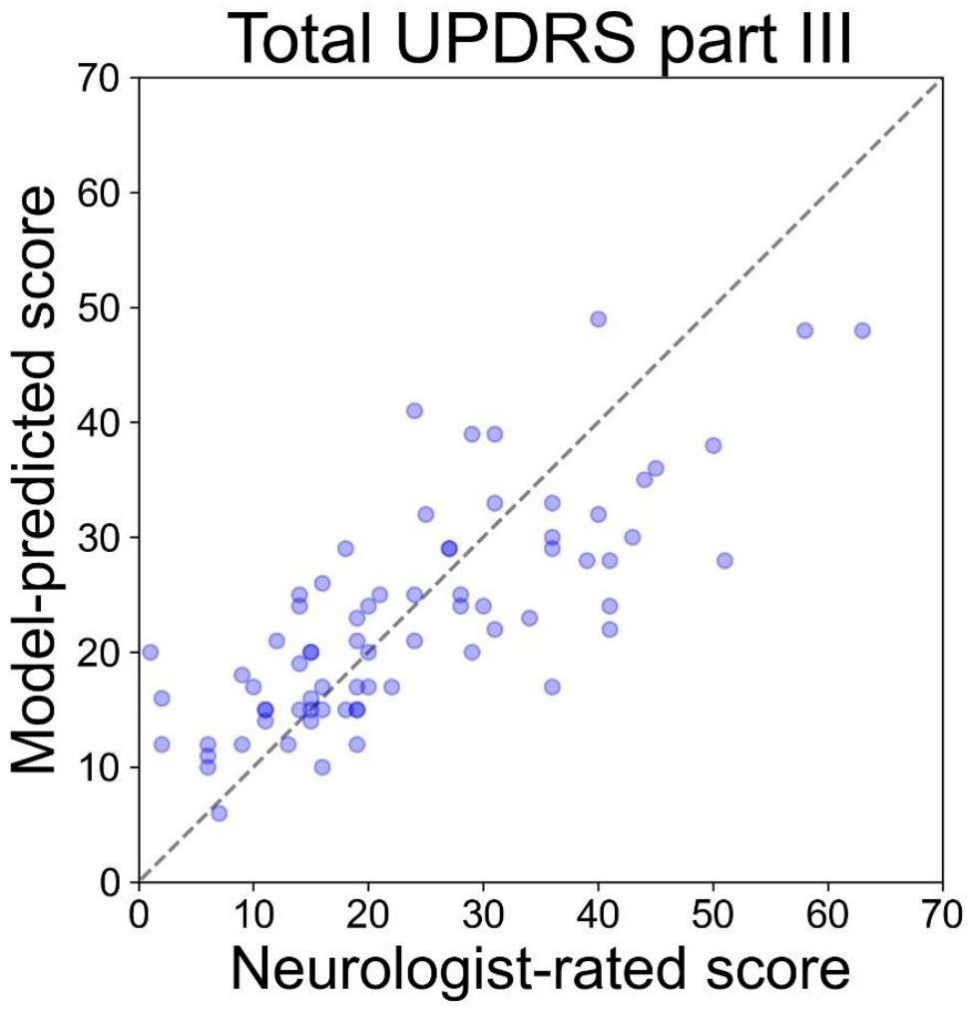
Scatter plots of neurologist-rated and CNN model-predicted scores for the total UPDRS part III score

The CNN model was first trained on 591 videos in the training dataset; thereafter, predictions of the 73 videos in the test dataset was evaluated as described previously. Figure 5 shows the scatter plots of the predicted total UPDRS part III scores in the test dataset. The MAE ± SD for the prediction of the scores for the total UPDRS part III in the test dataset was 6.9 ± 5.2, and the *R*^*2*^ value for these predictions was 0.59.

**Fig. 6:**
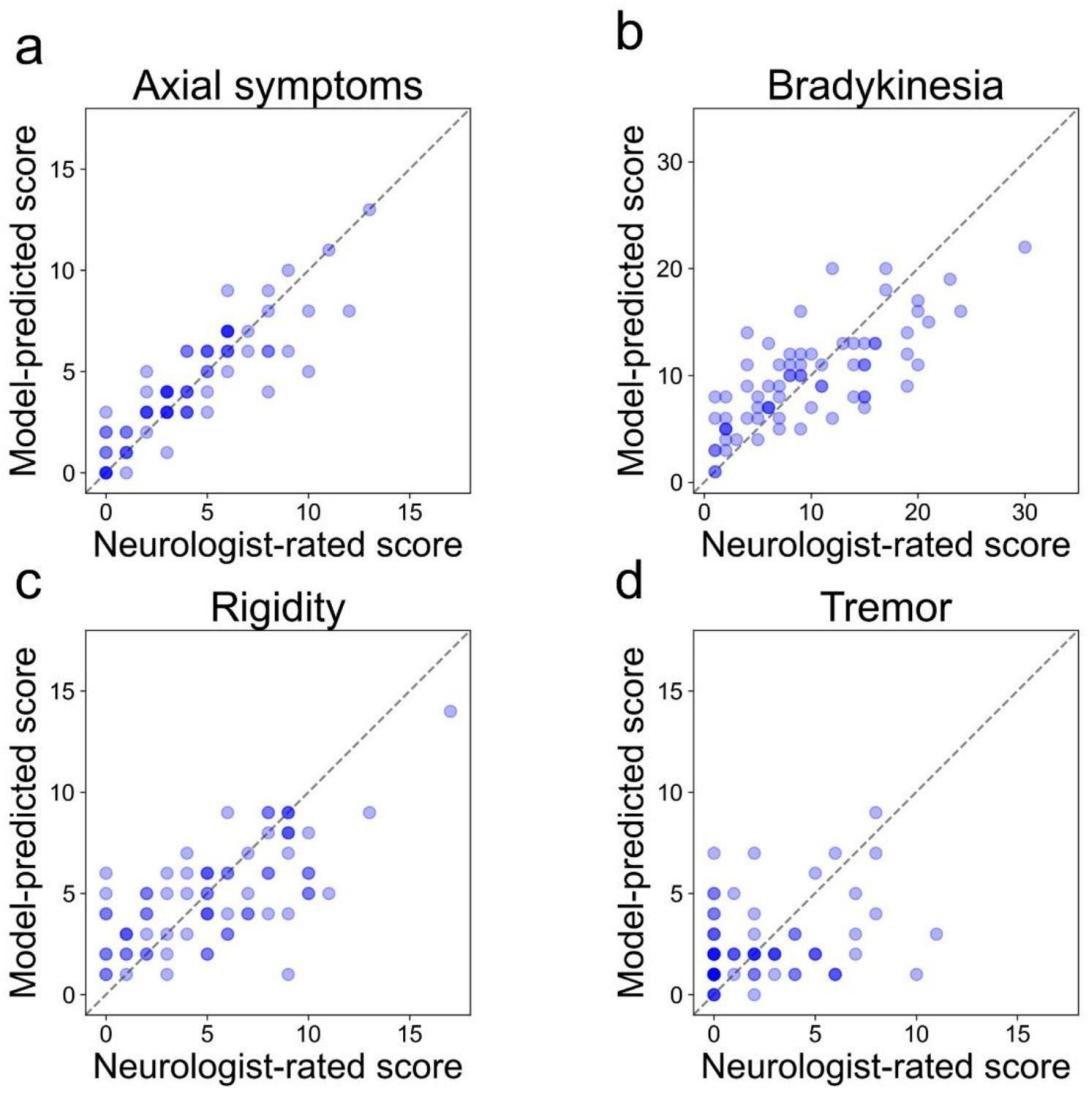
Scatter plots of neurologist-rated and CNN model-predicted subscores of UPDRS part III score. **(a)** Axial symptoms, **(b)** Bradykinesia, **(c)** Rigidity, **(d)** Tremor

Figure 6 demonstrates scatter plots of the predicted four subscores of UPDRS part III in the test dataset (a, axial symptoms; b, bradykinesia; c, rigidity; d, tremor). The MAE ± SD for the prediction of scores for axial symptoms, bradykinesia, rigidity, and tremor in the test dataset were 1.0 ± 1.1, 3.6 ± 2.6, 2.1 ± 1.7, and 2.1 ± 1.9, respectively. The corresponding *R*^*2*^ values for these predictions were 0.77, 0.56, 0.46, and 0.0, respectively. These results indicated that the model exhibited a more accurate predictive performance for the subscore of axial symptoms than for the other subscores.

Finally, we divided the videos of the test dataset into groups based on the neurologist-rated total UPDRS part III score in 10-point increments and compared the model performance for each group. The model performance for each group is described in Table 2. The MAE values were larger in predicting the scores of videos with higher UPDRS scores (more than 40) those of videos with low and moderate UPDRS scores. In the prediction of high UPDRS scores, the mean model-predicted scores were apparently lower than the mean neurologist-rated scores, suggesting that the model tended to underestimate the UPDRS scores in cases where the UPDRS scores were high.

**Table 2:** Model performance in predicting the total UPDRS part III score based on neurologist-rated UPDRS scores

Neurologist-rated UPDRS score	Number of videos	Mean neurologist-rated score ± SD	Mean model-predicted score ± SD	Mean absolute error ± SD
0–10	10	5.8 ± 3.2	13.4 ± 4.2	7.8 ± 5.4
11–20	29	16.0 ± 2.9	18.0 ± 4.7	4.2 ± 3.3
21–30	13	26.0 ± 2.9	27.0 ± 7.0	5.6 ± 4.4
31–40	11	35.5 ± 3.4	30.5 ± 8.6	8.5 ± 4.5
41–108	10	47.7 ± 9.1	33.7 ± 7.7	14.0 ± 4.6

UPDRS, Unified Parkinson’s Disease Rating Scale; SD, standard deviation

## Discussion

In this study, we assessed the feasibility of predicting the UPDRS part III score from patients’ gait videos by using a CNN model. Only a few studies have compared video-based (remote) and in-person assessments with the UPDRS. In one study conducted in a single institute in Spain with 44 patients with PD, the intraclass coefficient values between remote and in-person assessments with a modified version of the UPDRS part III were 0.53–0.68 at three intervals [16]. In a study including 11 participants in Australia, the median difference and interquartile range scores of a modified version of the UPDRS (sponsored by the Movement Disorder Society [17], MDS-UPDRS) between video-conference (remote) and in-person examinations were 3.0 and 1.5–9, respectively [18]. Compared with that in the abovementioned studies, our model’s predictive performance for the total UPDRS part III score seemed relatively low. A previous study that investigated the natural progression of the motor symptoms of PD reported that the UPDRS part III score in the drug-off state increased on average by 3.7 after 1 year [19]. The performance of our model seems too low to evaluate the natural progression of PD motor symptoms over 1 year. One of the potential reasons for its low performance is that the gait videos used in this study did not contain all aspects of PD motor symptoms. As the gait videos included limited information regarding patients’ speech, tremor, and muscle rigidity, it was difficult for the model to predict the total UPDRS part III score, as these aspects are also contained in the score. Therefore, we conducted an assessment to determine if the model could predict the UPDRS subscores of axial symptoms, bradykinesia, rigidity, and tremor. Among these subscores, the model displayed the highest performance in predicting axial symptoms, achieving an *R*^*2*^ value of 0.77. This high predictive performance might be attributed to the inclusion of items 28 (posture) and 29 (gait) in the axial symptoms subscore, which could be directly evaluated from the gait videos. However, the model exhibited relatively lower predictive performance for the bradykinesia and rigidity subscores, with *R*^*2*^ values of 0.56 and 0.44, respectively. The severity of bradykinesia was partially predictable from the gait speed, while the severity of rigidity was partially predictable from the arm swing. Nevertheless, the information available in the gait videos may not have been sufficient for accurate prediction of these subscores. The model failed to predict the tremor subscores, as indicated by an *R*^*2*^ value of 0. This could be attributed to the fact that the gait videos contained limited information on resting tremor involving the lower extremities or postural tremor, despite some patients exhibiting upper extremity tremor while walking. Additionally, the low sampling rate of frames in this model could have contributed to this issue. The frequency of resting tremor in typical Parkinson’s disease is 4–7 Hz [20]. However, in this study, we sampled 16 frames from a 10-second video and input them into the model, resulting in a sampling rate of 1.6 Hz. This sampling rate was too low to adequately capture resting tremor, suggesting that the model failed to predict the tremor subscore.

Another factor that may have affected the predictive performance for the total UPDRS part III is the limited amount of data, especially for patients with severe symptoms. Although the model performed well in predicting the UPDRS scores of patients with mild to moderate symptoms, the performance was low for videos that had been assigned high UPDRS scores by the neurologists. In all datasets, only 108 (14.7%) videos were assigned a score of > 40 for the total UPDRS part III score. Owing to limited data, the model was biased toward the prediction of values around the mean score and experienced difficulties in accurately evaluating patients with severe motor symptoms. Therefore, larger datasets, especially of patients with severe motor symptoms, would improve the performance of the model, and further studies will be required to resolve this issue.

We used the ECO-Lite architecture to analyze the gait videos. One of the major features of this model is that it initially performs two-dimensional convolution at the frame level to extract feature maps, followed by three-dimensional convolution on these maps. This strategy sets it apart from other three-dimensional convolutional neural network architectures [21, 22] and offers the advantage of being computationally less expensive while still demonstrating high performance in video classification tasks, all within a relatively short learning time [6]. Given these advantageous characteristics, we made the decision to utilize the ECO-Lite model for the current study.

The major strength of our study is our use of a CNN model to analyze video data for the direct prediction of a part of the UPDRS score, without performing feature extraction in advance. In most previous studies of video-based analysis of the severity of PD motor symptoms, researchers used the position of a certain body part, such as the joints and fingers, to calculate the movement of that body part using pose estimation algorithms [23–26]. It is difficult to compare the accuracy of the evaluation of PD symptoms obtained by our model and those of previous studies because different items of the UPDRS were evaluated. However, our model has several advantages. First, our model requires only static frames of gait videos as input to predict UPDRS scores. In previous studies [23–25], further signal processing had to be applied to the data obtained from pose estimation algorithms before the severity of PD symptoms could be evaluated. In contrast, our method does not require this additional feature extraction. Second, our model could be used to predict the cumulative score of multiple items of the UPDRS, including items other than that for gait (item 29). Previous models using pose estimation algorithms could predict the scores of only those items in the UPDRS part III relevant to the analysis of joints [27]. Our model, however, could be used to evaluate a broader range of PD symptoms and could be more useful in clinical settings. Although the model could predict only the part of the UPDRS score associated with PD symptoms such as bradykinesia, postural abnormality, and gait disturbance, our model is easy to use and could provide clinically useful information to evaluate PD symptoms.

An alternative solution to the remote assessment of PD symptoms is having patients wear sensing devices, such as accelerometers and gyroscopes [28]. There have been many studies in which PD symptoms, such as tremor, bradykinesia, postural instability, and gait impairment, were successfully assessed in this way [29]. However, sensor-based assessment of PD symptoms has several limitations. First, wearable devices are commercially available in only a few developed countries. Second, patients with PD are likely to be older individuals who may not be familiar with contemporary digital devices. Older individuals who cannot use wearable devices properly may not be included in studies on these devices, which may cause selection bias [28]. Conversely, our model uses only video data obtained from standard cameras, which are commonly accessible and easy to use. In contrast to sensor-based analysis, our method is difficult to use for the assessment of tremors. However, our model does not exclude the concurrent use of sensor-based analysis. Combining our model with wearable devices may allow the assessment of a broader range of PD symptoms.

This study has some limitations. A major limitation was that the number of participants was relatively small. In general, the performance of deep learning models can be enhanced by incorporating a larger amount of data for training. To address the limitation of having a small number of participants, we employed multiple videos from a single patient for training purposes and utilized a number of data augmentation techniques. However, the number of videos obtained varied greatly between patients. We accounted for this variance by randomly dividing all the video data and stratifying them based on the UPDRS score rather than on individual patients. Therefore, videos from the same patient were divided into training, validation, and test datasets. Although the UPDRS score varied even for the same patient with different medication states (medication on/off), DBS states (DBS on/off), and dates of video recording, the repeated use of videos from the same patient could have overestimated the final predictive performance of the model. Additionally, the use of repeated UPDRS ratings of the same patients could have created potential bias in the results. Second, we did not evaluate the inter-rater difference of the UPDRS scores between the two neurologists who assigned those scores because they did not assess the same patients at the same time; this could have also affected the result. Third, it is important to note that we did not strictly predetermine the walking distance during the gait video recording. This variation in walking distance might have influenced the predictive performance of the model. Fourth, we acknowledge that our assessment of the patient’s cognitive function was not exhaustive. Although we conducted the MMSE for most patients (except two) during the initial video recording, it is recognized that the MMSE may not be the most optimal instrument to detect cognitive impairment in PD [30]. Furthermore, for several patients, MMSE scores at follow-up assessments were not available, even though some patients were followed up for up to seven years. Therefore, cognitive decline could have potentially influenced gait patterns in some patients. To address these limitations, further studies with additional datasets divided based on individual patients, including new patients under standardized conditions with adequate cognitive assessment, are required to assess the external generalizability of the results. Fifth, because of the nature of the CNN, it is difficult to understand which information from the videos was used by our model to predict the UPDRS score. We speculate that gait features, such as walking speed, posture, arm swing, and freezing of gait, were used. However, elements in the videos that are irrelevant to PD symptoms (e.g., patients’ clothes, medical staff, and the background scene) could have influenced the predicted score. By using videos with various backgrounds, the model would learn that the background is not relevant to the UPDRS scores. Therefore, obtaining videos with various backgrounds through a multicenter study would contribute to the generalizability of the model. Sixth, it is important to note that this study did not include healthy controls, which hinders our ability to assess whether the model can accurately detect Parkinson’s disease symptoms in a population that includes healthy individuals. Finally, we could not assess the reproducibility of the model because we did not repeatedly record videos of the same participant on the same date and in the same treatment state. In further studies, the reproducibility of the model should be assessed.

## Conclusion

In this study, we aimed to determine the feasibility of predicting UPDRS scores from gait video data of patients with PD using a CNN model. While the model exhibited a low predictive performance for the total UPDRS part III score, it demonstrated relatively high performance in predicting subscores of axial symptoms. The result suggested that the model can capture aspects of PD symptoms, such as postural abnormality and gait disturbance, from gait videos and can thus be used to predict the UPDRS score associated with these symptoms. While the model’s predictive performance needs to be improved for use in clinical settings, we believe our model is a potential stepping-stone toward developing a computer-aided method for the evaluation of PD symptoms from patient videos. This kind of method will contribute to reducing patients’ burden of visiting the clinic and their exposure to diseases during the pandemic era.

### Electronic supplementary material

Below is the link to the electronic supplementary material.

**Additional file 1**: Supporting Table S1. Demographic data for each patient (N = 74). Demographic data for each patient and the MMSE and LEDD scores for each patient.

**Additional file 2**: Supporting Table S2. Number of videos and the Unified Parkinson’s Disease Rating Scale scores for each patient (N = 74). Number of videos and the total UPDRS score for each patient, as well as the tremor, rigidity, bradykinesia, and axial subscores.

## Data Availability

The video data are not available for public access because of patient privacy concerns. All other anonymized data can be provided by the corresponding author on reasonable request.

## Abbreviations

CNN: Convolutional neural network
DBS: Deep brain stimulation
LEDD: Levodopa equivalent daily dose
lr: Learning rate
MAE: Mean absolute error
PD: Parkinson’s disease
*R*^*2*^: Coefficient of determination
SD: Standard deviation
UPDRS: Unified Parkinson’s Disease Rating Scale

## References

[1] Nussbaum RL, Ellis CE (2003). Alzheimer’s disease and Parkinson’s disease. N Engl J Med.

[2] Kalia LV, Lang AE (2015). Parkinson’s disease. Lancet.

[3] Bloem BR, Dorsey ER, Okun MS (2020). The coronavirus disease 2019 crisis as catalyst for telemedicine for chronic neurological disorders. JAMA Neurol.

[4] Ohannessian R, Duong TA, Odone A (2020). Global telemedicine implementation and integration within health systems to fight the COVID-19 pandemic: a call to action. JMIR Public Health Surveill.

[5] Kidziński Ł, Yang B, Hicks JL, Rajagopal A, Delp SL, Schwartz MH (2020). Deep neural networks enable quantitative movement analysis using single-camera videos. Nat Commun.

[6] Zolfaghari M, Singh K, Brox T. ECO: efficient convolutional network for online video understanding. Lecture Notes in Computer Science. Proceedings of the European Conference on Computer Vision (ECCV). 2018:713 – 30.

[7] Martínez-Martín P, Gil-Nagel A, Gracia LM, Gómez JB, Martínez-Sarriés J, Bermejo F (1994). Unified Parkinson’s Disease Rating Scale characteristics and structure. The Cooperative Multicentric Group. Mov Disord.

[8] Hughes AJ, Daniel SE, Kilford L, Lees AJ (1992). Accuracy of clinical diagnosis of idiopathic Parkinson’s disease: a clinico-pathological study of 100 cases. J Neurol Neurosurg Psychiatry.

[9] Litvan I, Bhatia KP, Burn DJ, Goetz CG, Lang AE, McKeith I (2003). Movement Disorders Society scientific issues committee report: SIC Task Force appraisal of clinical diagnostic criteria for parkinsonian disorders. Mov Disord.

[10] Tomlinson CL, Stowe R, Patel S, Rick C, Gray R, Clarke CE (2010). Systematic review of levodopa dose equivalency reporting in Parkinson’s disease. Mov Disord.

[11] Schade S, Mollenhauer B, Trenkwalder C (2020). Levodopa equivalent dose conversion factors: an updated proposal including opicapone and safinamide. Mov Disord Clin Pract.

[12] Kay W, Carreira J, Simonyan K, Zhang B, Hillier C, Vijayanarasimhan S (2017).

[13] Ioffe S, Szegedy C. Batch normalization: accelerating deep network training by reducing internal covariate shift. Presented at the 32nd International Conference on Machine Learning, Lille, France; vol. 2015; 2015.

[14] Tran D, Ray J, Shou Z, Chang SF, Paluri M (2017). ConvNet architecture search for spatiotemporal feature learning. arXiv.

[15] Paszke A, Gross S, Massa F, Lerer A, Bradbury J, Chanan G (2019). PyTorch: an imperative style, high-performance deep learning library. arXiv.

[16] Cubo E, Gabriel-Galán JMT, Martínez JS, Alcubilla CR, Yang C, Arconada OF (2012). Comparison of office-based versus home web-based clinical assessments for Parkinson’s disease. Mov Disord.

[17] Goetz CG, Tilley BC, Shaftman SR, Stebbins GT, Fahn S, Martinez-Martin P (2008). Movement Disorder Society-Sponsored revision of the Unified Parkinson’s Disease Rating Scale (MDS-UPDRS): scale presentation and clinimetric testing results. Mov Disord.

[18] Stillerova T, Liddle J, Gustafsson L, Lamont R, Silburn P (2016). Remotely assessing symptoms of Parkinson’s disease using videoconferencing: a feasibility study. Neurol Res Int.

[19] Cilia R, Cereda E, Akpalu A, Sarfo FS, Cham M, Laryea R (2020). Natural history of motor symptoms in Parkinson’s disease and the long-duration response to levodopa. Brain.

[20] Baumann CR (2012). Epidemiology, diagnosis and differential diagnosis in Parkinson’s disease tremor. Parkinsonism Relat Disord.

[21] Tran D, Bourdev L, Fergus R, Torresani L, Paluri M. Learning spatiotemporal features with 3d convolutional networks. Proceedings of the I.E.E.E. International Conference on Computer Vision. 2015:4489-97.

[22] Carreira J, Andrew Z. Quo vadis, action recognition? a new model and the kinetics dataset. Proceedings of the IEEE Conference on Computer Vision and Pattern Recognition. 2017:6299 – 308.

[23] Li MH, Mestre TA, Fox SH, Taati B (2018). Vision-based assessment of parkinsonism and levodopa-induced dyskinesia with pose estimation. J Neuroeng Rehabil.

[24] Sato K, Nagashima Y, Mano T, Iwata A, Toda T (2019). Quantifying normal and parkinsonian gait features from home movies: practical application of a deep learning-based 2D pose estimator. PLoS ONE.

[25] Shin JH, Ong JN, Kim R, Park SM, Choi J, Kim HJ (2020). Objective measurement of limb bradykinesia using a marker-less tracking algorithm with 2D-video in PD patients. Parkinsonism Relat Disord.

[26] Park KW, Lee EJ, Lee JS, Jeong J, Choi N, Jo S (2021). Machine learning-based automatic rating for cardinal symptoms of Parkinson disease. Neurology.

[27] Silva de Lima AL, Smits T, Darweesh SKL, Valenti G, Milosevic M, Pijl M (2020). Home-based monitoring of falls using wearable sensors in Parkinson’s disease. Mov Disord.

[28] Rovini E, Maremmani C, Cavallo F (2017). How wearable sensors can support Parkinson’s disease diagnosis and treatment: a systematic review. Front Neurosci.

[29] Kubota KJ, Chen JA, Little MA (2016). Machine learning for large-scale wearable sensor data in Parkinson’s disease: concepts, promises, pitfalls, and futures. Mov Disord.

[30] Hoops S, Nazem S, Siderowf AD, Duda JE, Xie SX, Stern MB (2009). Validity of the MoCA and MMSE in the detection of MCI and dementia in Parkinson disease. Neurology.

