# Supplementary material for: Gait video-based prediction of unified Parkinson’s disease rating scale score: a retrospective study
Source: BMC Neurol. 2023 Oct 5;23:358. doi: 10.1186/s12883-023-03385-2 (PMC10552271; doi:10.1186/s12883-023-03385-2)
Supplement: Supplementary file 1 — Additional file 1: Supporting Table S1. Demographic data for each patient (N = 74). Demographic data for each patient and the MMSE and LEDD scores for each patient. [file 12883_2023_3385_MOESM1_ESM.docx]

Supporting Table S1. Demographic data for each patient (N=74)

| Patient | Sex | Age | Disease duration | Duration between  first and last  video assessments | DAT | HY stage  (medication on) | HY stage  (medication off) | MMSE  score | LEDD  score |
| --- | --- | --- | --- | --- | --- | --- | --- | --- | --- |
| 1 | M | 49 | 8 | 79 | Bil. STN-DBS | 2 | 3 | 30 | 416 |
| 2 | F | 76 | 18 | 45 | Bil. STN-DBS | 2 | 5 | 30 | 825 |
| 3 | F | 67 | 23 | 61 | Bil. STN-DBS | 2 | 5 | 27 | 1077 |
| 4 | F | 62 | 8 | 66 | Bil. STN-DBS | 3 | 5 | 28 | 669 |
| 5 | F | 71 | 9 | 62 | Bil. STN-DBS | 2 | 2 | 30 | 549 |
| 6 | F | 51 | 9 | 67 | Lt. PSA-DBS | 2 | 2 | 30 | 225 |
| 7 | F | 54 | 13 | 73 | Bil. STN-DBS | 4 | 4 | 30 | 652 |
| 8 | F | 67 | 13 | 54 | Bil. STN-DBS | 4 | 4 | 29 | 765 |
| 9 | F | 76 | 21 | 7 | Bil. GPi-DBS | 4 | 5 | 26 | 679 |
| 10 | F | 63 | 14 | 67 | Bil. STN-DBS | 3 | 4 | 29 | 1045 |
| 11 | F | 50 | 13 | 64 | Bil. STN-DBS | 2 | 5 | 27 | 1035 |
| 12 | F | 71 | 14 | 54 | Bil. PSA-DBS | 3 | 5 | 25 | 2155 |
| 13 | M | 69 | 2 | 53 | Lt. STN-DBS | 2 | 2 | 28 | 150 |
| 14 | F | 73 | 15 | 52 | Bil. STN-DBS | 3 | 4 | 28 | 552 |
| 15 | F | 56 | 6 | 56 | Bil. STN-DBS | 2 | 4 | 29 | 798 |
| 16 | M | 66 | 11 | 51 | Bil. STN-DBS | 2 | 5 | 28 | 988 |
| 17 | M | 73 | 1 | 27 | Bil. PSA-DBS | 2 | 2 | 30 | 100 |
| 18 | F | 69 | 17 | 43 | Rt. STN-DBS | 3 | 5 | 30 | 678 |
| 19 | F | 60 | 8 | 42 | Bil. STN-DBS | 2 | 3 | 27 | 688 |
| 20 | M | 50 | 13 | 30 | Bil. PSA-DBS | 2 | 4 | 30 | 1052 |
| 21 | F | 59 | 8 | 9 | Bil. STN-DBS | 1 | 3 | 29 | 978 |
| 22 | F | 72 | 10 | 6 | Bil. STN-DBS | 4 | 5 | 28 | 1191 |
| 23 | M | 57 | 17 | 18 | Bil. STN-DBS | 2 | 5 | 25 | 1141 |
| 24 | F | 58 | 7 | 15 | Bil. STN-DBS | 3 | 5 | 26 | 1410 |
| 25 | M | 68 | 17 | 18 | Bil. STN-DBS | 3 | 4 | 29 | 1460 |
| 26 | F | 57 | 16 | 16 | Bil. STN-DBS | 3 | 4 | 26 | 1528 |
| 27 | F | 67 | 13 | 1 | Bil. STN-DBS | 2 | 4 | 27 | 1004 |
| 28 | F | 69 | 8 | 10 | Bil. STN-DBS | 3 | 4 | 30 | 899 |
| 29 | M | 55 | 11 | 10 | Bil. STN-DBS | 3 | 4 | 28 | 1262 |
| 30 | M | 76 | 22 | 1 | Bil. STN-DBS | 3 | 4 | 23 | 696 |
| 31 | F | 65 | 20 | 1 | Bil. PSA-DBS | 3 | 4 | 21 | 817 |
| 32 | M | 74 | 23 | 11 | Lt. PSA-DBS | 4 | 4 | 29 | 610 |
| 33 | F | 73 | 9 | 1 | - | 3 | 5 | 25 | 1837 |
| 34 | F | 59 | 14 | 1 | Bil. PSA-DBS | 3 | 3 | 30 | 530 |
| 35 | M | 70 | 15 | 1 | Bil. PSA-DBS | 2 | 4 | 22 | 550 |
| 36 | F | 66 | 16 | 1 | Bil. PSA-DBS | 3 | 4 | 25 | 641 |
| 37 | F | 60 | 15 | 1 | Bil. STN-DBS | 3 | 5 | 27 | 692 |
| 38 | F | 70 | 4 | 30 | - | 2 | 3 | 29 | 279 |
| 39 | F | 76 | 12 | 1 | - | 3 | 5 | 29 | 722 |
| 40 | M | 73 | 9 | 1 | - | 2 | 2 | 28 | 388 |
| 41 | F | 71 | 10 | 1 | - | 2 | 4 | 25 | 798 |
| 42 | M | 69 | 10 | 1 | - | 2 | 4 | 28 | 990 |
| 43 | M | 66 | 1 | 1 | - | 4 | 4 | -^a^ | 0 |
| 44 | M | 66 | 10 | 26 | - | 3 | 4 | 29 | 999 |
| 45 | M | 61 | 13 | 1 | - | 2 | 4 | 25 | 1321 |
| 46 | F | 68 | 14 | 24 | Bil. STN-DBS | 3 | 3 | 30 | 300 |
| 47 | F | 70 | 10 | 1 | - | 3 | 5 | 28 | 885 |
| 48 | M | 53 | 2 | 1 | - | 2 | 2 | 28 | 375 |
| 49 | M | 43 | 18 | 1 | - | 2 | 5 | 30 | 1611 |
| 50 | F | 64 | 9 | 1 | LCIG | 4 | 5 | 28 | 677 |
| 51 | F | 68 | 12 | 1 | - | 4 | 4 | 25 | 1049 |
| 52 | F | 69 | 11 | 1 | - | 4 | 5 | 26 | 1294 |
| 53 | M | 35 | 4 | 1 | - | 2 | 2 | 28 | 594 |
| 54 | M | 60 | 9 | 1 | - | 1 | 5 | 30 | 2013 |
| 55 | M | 58 | 8 | 1 | - | 2 | 4 | 27 | 1207 |
| 56 | M | 53 | 13 | 1 | - | 2 | 2 | 30 | 490 |
| 57 | F | 68 | 16 | 1 | Bil. STN-DBS | 4 | 4 | -^a^ | 539 |
| 58 | F | 57 | 8 | 1 | - | 2 | 2 | 30 | 664 |
| 59 | M | 58 | 11 | 1 | - | 2 | 2 | 28 | 250 |
| 60 | F | 62 | 13 | 1 | LCIG | 4 | 5 | 29 | 1166 |
| 61 | M | 51 | 16 | 1 | - | 2 | 2 | 28 | 1219 |
| 62 | F | 59 | 4 | 6 | Lt. PSA-DBS | 1 | 2 | 30 | 450 |
| 63 | M | 67 | 8 | 6 | Bil. STN-DBS | 2 | 2 | 27 | 720 |
| 64 | M | 67 | 8 | 1 | - | 3 | 4 | 26 | 483 |
| 65 | M | 59 | 6 | 1 | - | 3 | 3 | 28 | 1513 |
| 66 | F | 69 | 4 | 1 | - | 2 | 3 | 28 | 300 |
| 67 | F | 61 | 10 | 1 | - | 2 | 5 | 29 | 1410 |
| 68 | F | 64 | 11 | 2 | Bil. STN-DBS | 2 | 3 | 29 | 988 |
| 69 | M | 71 | 13 | 1 | - | 2 | 3 | 30 | 1404 |
| 70 | F | 59 | 14 | 8 | Bil. STN-DBS | 2 | 3 | 30 | 560 |
| 71 | F | 68 | 3 | 1 | - | 3 | 3 | 30 | 300 |
| 72 | F | 68 | 17 | 1 | - | 2 | 2 | 27 | 600 |
| 73 | M | 55 | 11 | 1 | - | 2 | 2 | 29 | 915 |
| 74 | M | 58 | 9 | 1 | - | 2 | 5 | 30 | –^b^ |

M, male; F, female; HY stage, Hoehn and Yahr stage; MMSE, Mini-Mental State Examination; LEDD, levodopa equivalent daily dose; UPDRS, Unified Parkinson’s Disease Rating Scale; DAT, device-aided therapy; SD, standard deviation; DBS, deep brain stimulation; LCIG; levodopa-carbidopa intestinal gel; STN, subthalamic nucleus; PSA, posterior subthalamic area; GPi, globus pallidus interna; Bil, bilateral; lt, left; rt, right.

Age, disease duration, HY stages, and scores of MMSE were obtained at the time of the first video assessment.

^a^Patients 43 and 57 were not assessed via the MMSE.

^b^The LEDD of patient 74 was not calculated because the patient used a ropinirole patch.
