# Supplementary material for: Gait video-based prediction of unified Parkinson’s disease rating scale score: a retrospective study
Source: BMC Neurol. 2023 Oct 5;23:358. doi: 10.1186/s12883-023-03385-2 (PMC10552271; doi:10.1186/s12883-023-03385-2)
Supplement: Supplementary file 2 — Additional file 2: Supporting Table S2. Number of videos and the Unified Parkinson’s Disease Rating Scale scores for each patient (N = 74). Number of videos and the total UPDRS score for each patient, as well as the tremor, rigidity, bradykinesia, and axial subscores. [file 12883_2023_3385_MOESM2_ESM.docx]

Supporting Table S2. Number of videos and the Unified Parkinson’s Disease Rating Scale scores for each patient (N=74)

| Patient | Number of videos | Total UPDRS part III score | Tremor subscore | Rigidity subscore | Bradykinesia subscore | Axial symptoms subscore |
| --- | --- | --- | --- | --- | --- | --- |
| 1 | 34 | 23.1 ± 12.5 | 3.5 ± 3.1 | 3.2 ± 2.4 | 10.9 ± 6.4 | 4.4 ± 1.8 |
| 2 | 18 | 21.2 ± 14.1 | 2.7 ± 3.4 | 3.1 ± 3.1 | 7.6 ± 5.7 | 5.4 ± 3.4 |
| 3 | 36 | 18.1 ± 10.7 | 2.1 ± 2.8 | 3.9 ± 3.4 | 6.4 ± 4.6 | 4.2 ± 2.3 |
| 4 | 28 | 15.0 ± 10.0 | 0.0 ± 0.2 | 2.8 ± 3.0 | 8.2 ± 5.7 | 3.6 ± 2.3 |
| 5 | 23 | 23.3 ± 7.6 | 3.7 ± 2.8 | 6.3 ± 2.0 | 11.0 ± 2.5 | 0.9 ± 1.3 |
| 6 | 28 | 18.1 ± 6.2 | 2.9 ± 2.5 | 6.5 ± 1.9 | 6.4 ± 3.0 | 1.0 ± 0.7 |
| 7 | 30 | 28.3 ± 14.6 | 1.4 ± 1.8 | 6.0 ± 3.9 | 13.6 ± 6.9 | 4.8 ± 2.2 |
| 8 | 20 | 21.3 ± 8.5 | 0.9 ± 1.0 | 2.8 ± 3.0 | 9.4 ± 3.8 | 6.1 ± 2.3 |
| 9 | 2 | 28.5 ± 13.4 | 0.0 ± 0.0 | 5.0 ± 1.4 | 12.5 ± 9.2 | 8.0 ± 1.4 |
| 10 | 23 | 32.7 ± 9.3 | 1.7 ± 2.6 | 8.8 ± 1.8 | 13.0 ± 5.6 | 6.0 ± 2.0 |
| 11 | 26 | 15.4 ± 14.5 | 2.1 ± 4.1 | 2.2 ± 2.5 | 5.9 ± 5.9 | 3.0 ± 2.9 |
| 12 | 22 | 31.8 ± 12.2 | 5.0 ± 5.0 | 4.5 ± 2.7 | 10.6 ± 4.8 | 7.5 ± 2.4 |
| 13 | 32 | 12.4 ± 4.6 | 3.0 ± 2.5 | 4.4 ± 1.5 | 3.4 ± 2.9 | 0.7 ± 0.7 |
| 14 | 30 | 33.9 ± 11.2 | 3.5 ± 3.5 | 7.9 ± 2.8 | 15.1 ± 5.5 | 5.1 ± 1.9 |
| 15 | 27 | 21.1 ± 11.0 | 1.4 ± 2.1 | 3.1 ± 1.8 | 9.4 ± 5.1 | 4.1 ± 2.8 |
| 16 | 28 | 15.6 ± 6.4 | 0.0 ± 0.0 | 3.3 ± 2.3 | 4.7 ± 2.8 | 5.3 ± 2.6 |
| 17 | 22 | 28.7 ± 8.3 | 3.0 ± 3.0 | 8.3 ± 2.1 | 14.4 ± 4.2 | 0.5 ± 0.8 |
| 18 | 23 | 36.7 ± 15.7 | 2.1 ± 2.3 | 10.4 ± 3.4 | 14.7 ± 7.7 | 7.7 ± 3.5 |
| 19 | 18 | 24.1 ± 17.0 | 3.0 ± 2.6 | 3.1 ± 2.4 | 10.1 ± 6.6 | 5.9 ± 5.7 |
| 20 | 22 | 31.0 ± 21.2 | 3.1 ± 4.1 | 7.0 ± 3.0 | 13.7 ± 11.2 | 4.0 ± 4.0 |
| 21 | 10 | 18.4 ± 7.7 | 0.4 ± 0.8 | 3.4 ± 2.2 | 7.1 ± 3.1 | 6.3 ± 3.5 |
| 22 | 8 | 27.1 ± 11.1 | 2.8 ± 1.5 | 4.2 ± 3.3 | 12.0 ± 2.9 | 6.1 ± 4.1 |
| 23 | 17 | 20.2 ± 10.7 | 0.6 ± 0.9 | 4.4 ± 3.7 | 9.5 ± 6.9 | 2.3 ± 1.0 |
| 24 | 18 | 18.6 ± 7.3 | 2.1 ± 1.8 | 2.1 ± 2.0 | 8.4 ± 2.1 | 3.7 ± 2.4 |
| 25 | 18 | 14.9 ± 7.0 | 1.3 ± 0.8 | 3.4 ± 2.6 | 4.1 ± 2.7 | 5.1 ± 1.7 |
| 26 | 18 | 36.1 ± 19.1 | 0.1 ± 0.5 | 3.1 ± 3.1 | 23.0 ± 11.7 | 8.1 ± 3.9 |
| 27 | 2 | 26.0 ± 9.9 | 1.5 ± 0.7 | 3.5 ± 0.7 | 13.5 ± 4.9 | 4.0 ± 2.8 |
| 28 | 10 | 31.3 ± 12.8 | 0.0 ± 0.0 | 6.3 ± 3.0 | 14.5 ± 5.4 | 7.6 ± 5.1 |
| 29 | 12 | 28.8 ± 9.8 | 1.2 ± 1.2 | 9.2 ± 3.3 | 9.9 ± 5.2 | 5.8 ± 0.8 |
| 30 | 4 | 30.8 ± 8.7 | 2.5 ± 3.7 | 5.5 ± 2.4 | 13.5 ± 2.9 | 5.5 ± 0.6 |
| 31 | 4 | 18.2 ± 3.6 | 0.0 ± 0.0 | 1.0 ± 1.2 | 7.8 ± 1.5 | 7.5 ± 1.3 |
| 32 | 12 | 37.2 ± 11.0 | 5.6 ± 1.8 | 5.9 ± 3.2 | 13.5 ± 5.8 | 7.8 ± 1.5 |
| 33 | 2 | 27.5 ± 0.7 | 0.5 ± 0.7 | 8.0 ± 0.0 | 11.0 ± 1.4 | 5.0 ± 0.0 |
| 34 | 2 | 18.5 ± 2.1 | 0.0 ± 0.0 | 6.0 ± 0.0 | 5.0 ± 1.4 | 7.0 ± 0.0 |
| 35 | 4 | 33.0 ± 12.4 | 6.5 ± 5.9 | 5.8 ± 1.7 | 14.8 ± 5.4 | 4.0 ± 0.8 |
| 36 | 2 | 21.0 ± 5.7 | 2.5 ± 3.5 | 3.0 ± 1.4 | 6.5 ± 0.7 | 7.0 ± 0.0 |
| 37 | 2 | 37.5 ± 12.0 | 0.0 ± 0.0 | 0.0 ± 0.0 | 23.5 ± 9.2 | 11.5 ± 0.7 |
| 38 | 5 | 13.0 ± 1.4 | 1.4 ± 1.1 | 4.6 ± 0.9 | 2.4 ± 0.9 | 3.2 ± 1.1 |
| 39 | 1 | 19.0 ± 0 | 0.0 ± 0 | 8.0 ± 0 | 6.0 ± 0 | 3.0 ± 0 |
| 40 | 2 | 16.5 ± 0.7 | 4.0 ± 0.0 | 7.5 ± 2.1 | 3.0 ± 1.4 | 1.0 ± 1.4 |
| 41 | 2 | 36.5 ± 7.8 | 1.0 ± 1.4 | 10.5 ± 0.7 | 16.5 ± 2.1 | 6.0 ± 4.2 |
| 42 | 3 | 23.7 ± 13.7 | 2.7 ± 2.5 | 7.3 ± 4.6 | 9.0 ± 5.6 | 3.3 ± 3.2 |
| 43 | 2 | 25.0 ± 2.8 | 0.0 ± 0.0 | 6.5 ± 2.1 | 13.5 ± 0.7 | 4.0 ± 0.0 |
| 44 | 4 | 30.5 ± 10.5 | 0.0 ± 0.0 | 5.0 ± 2.2 | 13.2 ± 7.3 | 9.2 ± 2.1 |
| 45 | 2 | 22.0 ± 12.7 | 0.0 ± 0.0 | 2.5 ± 3.5 | 13.5 ± 4.9 | 2.5 ± 2.1 |
| 46 | 12 | 33.1 ± 9.7 | 2.5 ± 2.9 | 5.1 ± 2.6 | 16.8 ± 4.4 | 4.8 ± 2.5 |
| 47 | 2 | 15.0 ± 7.1 | 0.0 ± 0.0 | 2.5 ± 0.7 | 6.5 ± 4.9 | 5.0 ± 0.0 |
| 48 | 2 | 16.0 ± 7.1 | 1.0 ± 0.0 | 5.5 ± 2.1 | 6.5 ± 3.5 | 0.5 ± 0.7 |
| 49 | 2 | 35.5 ± 38.9 | 1.5 ± 0.7 | 8.5 ± 7.8 | 18.0 ± 21.2 | 5.0 ± 7.1 |
| 50 | 1 | 24.0 ± 0 | 0.0 ± 0 | 2.0 ± 0 | 10.0 ± 0 | 10.0 ± 0 |
| 51 | 2 | 32.5 ± 7.8 | 1.0 ± 1.4 | 7.0 ± 2.8 | 14.0 ± 2.8 | 9.0 ± 1.4 |
| 52 | 2 | 52.5 ± 13.4 | 6.0 ± 4.2 | 10.0 ± 2.8 | 20.5 ± 2.1 | 12.0 ± 2.8 |
| 53 | 2 | 16.0 ± 15.6 | 1.5 ± 2.1 | 5.0 ± 1.4 | 6.5 ± 9.2 | 0.5 ± 0.7 |
| 54 | 2 | 23.0 ± 18.4 | 0.0 ± 0.0 | 7.0 ± 2.8 | 9.5 ± 10.6 | 3.5 ± 4.9 |
| 55 | 2 | 25.0 ± 25.5 | 0.0 ± 0.0 | 6.0 ± 7.1 | 9.5 ± 9.2 | 7.5 ± 7.8 |
| 56 | 2 | 15.0 ± 7.1 | 0.0 ± 0.0 | 3.0 ± 1.4 | 9.5 ± 3.5 | 1.5 ± 0.7 |
| 57 | 2 | 28.0 ± 5.7 | 0.0 ± 0.0 | 3.5 ± 3.5 | 12.0 ± 1.4 | 8.0 ± 0.0 |
| 58 | 3 | 27.3 ± 3.8 | 3.3 ± 1.2 | 5.7 ± 0.6 | 12.3 ± 0.6 | 4.0 ± 1.7 |
| 59 | 1 | 13.0 ± 0 | 4.0 ± 0 | 4.0 ± 0 | 2.0 ± 0 | 2.0 ± 0 |
| 60 | 1 | 19.0 ± 0 | 0.0 ± 0 | 2.0 ± 0 | 7.0 ± 0 | 8.0 ± 0 |
| 61 | 2 | 15.0 ± 4.2 | 1.5 ± 2.1 | 4.0 ± 0.0 | 7.0 ± 1.4 | 0.0 ± 0.0 |
| 62 | 6 | 11.7 ± 4.1 | 5.5 ± 3.4 | 1.5 ± 0.8 | 4.5 ± 1.4 | 0.2 ± 0.4 |
| 63 | 7 | 14.9 ± 3.8 | 0.4 ± 0.5 | 2.3 ± 1.1 | 8.3 ± 1.8 | 2.1 ± 1.2 |
| 64 | 2 | 26.0 ± 5.7 | 0.0 ± 0.0 | 5.0 ± 1.4 | 12.0 ± 1.4 | 5.0 ± 2.8 |
| 65 | 2 | 31.5 ± 7.8 | 0.5 ± 0.7 | 8.5 ± 0.7 | 14.5 ± 4.9 | 4.0 ± 1.4 |
| 66 | 2 | 30.0 ± 8.5 | 5.0 ± 1.4 | 7.5 ± 2.1 | 13.0 ± 2.8 | 2.0 ± 1.4 |
| 67 | 2 | 25.5 ± 23.3 | 1.0 ± 1.4 | 4.5 ± 3.5 | 8.5 ± 7.8 | 9.0 ± 8.5 |
| 68 | 4 | 16.8 ± 7.0 | 0.0 ± 0.0 | 2.0 ± 2.7 | 10.5 ± 3.0 | 1.8 ± 1.5 |
| 69 | 2 | 15.5 ± 3.5 | 0.5 ± 0.7 | 3.0 ± 0.0 | 5.5 ± 0.7 | 4.5 ± 0.7 |
| 70 | 6 | 16.8 ± 6.9 | 0.0 ± 0.0 | 3.5 ± 2.6 | 8.5 ± 3.6 | 2.8 ± 1.3 |
| 71 | 1 | 17.0 ± 0 | 0.0 ± 0 | 3.0 ± 0 | 9.0 ± 0 | 3.0 ± 0 |
| 72 | 2 | 21.0 ± 7.1 | 8.5 ± 0.7 | 4.0 ± 2.8 | 6.0 ± 4.2 | 1.5 ± 0.7 |
| 73 | 2 | 22.0 ± 7.1 | 0.0 ± 0.0 | 2.5 ± 2.1 | 15.5 ± 3.5 | 1.0 ± 1.4 |
| 74 | 1 | 10.0 ± 0 | 0.0 ± 0 | 0.0 ± 0 | 9.0 ± 0 | 0.0 ± 0 |

UPDRS, Unified Parkinson’s Disease Rating Scale.
